# Exploring the impact of gut microbiota and diet on breast cancer risk and progression

**DOI:** 10.1002/ijc.33496

**Published:** 2021-02-12

**Authors:** Nancy M. Y. Teng, Christopher A. Price, Alastair M. McKee, Lindsay J. Hall, Stephen D. Robinson

**Affiliations:** ^1^ Gut Microbes & Health Quadram Institute Bioscience, Norwich Research Park Norwich UK; ^2^ Norwich Medical School University of East Anglia, Norwich Research Park Norwich UK; ^3^ Chair of Intestinal Microbiome, School of Life Sciences, ZIEL‐Institute for Food & Health Technical University of Munich Freising Germany; ^4^ School of Biological Sciences University of East Anglia, Norwich Research Park Norwich UK

**Keywords:** antibiotics, breast cancer, diet, immune, microbiota

## Abstract

There is emerging evidence that resident microbiota communities, that is, the microbiota, play a key role in cancer outcomes and anticancer responses. Although this has been relatively well studied in colorectal cancer and melanoma, other cancers, such as breast cancer (BrCa), have been largely overlooked to date. Importantly, many of the environmental factors associated with BrCa incidence and progression are also known to impact the microbiota, for example, diet and antibiotics. Here, we explore BrCa risk factors from large epidemiology studies and microbiota associations, and more recent studies that have directly profiled BrCa patients' gut microbiotas. We also discuss how in vivo studies have begun to unravel the immune mechanisms whereby the microbiota may influence BrCa responses, and finally we examine how diet and specific nutrients are also linked to BrCa outcomes. We also consider future research avenues and important considerations with respect to study design and implementation, and we highlight some of the important unresolved questions, which currently limit our overall understanding of the mechanisms underpinning microbiota‐BrCa responses.

**Abbreviations**
BrCa: breast cancer
CRC: colorectal cancer
HFD: high‐fat diet
LPS, lipopolysaccharide; MDSC: myeloid‐derived suppressor cells
SCFA: short‐chain fatty acid
TNBC: triple‐negative breast cancer

## 1 INTRODUCTION

Annually, breast cancer (BrCa) is predicted to affect over 2 million new patients, with more than 600 000 BrCa‐related deaths worldwide, second only to lung cancer in incidence and mortality.^1^ The financial implications of the disease on patients and health services are equally staggering with average treatment costs ranging from ~£22 000 to £115 000 for a single patient depending on disease stage.^2^ Moreover, while patients diagnosed in early stages usually have good prognostic outcomes, those diagnosed in late stages of the disease have very poor 5‐year survival rates, less than 30%.^2^ Thus, understanding the factors that drive BrCa development and progression is important not only for patient outcomes but also for alleviating financial burdens on healthcare systems.

BrCa is an extremely heterogeneous disease and thus traditional therapeutic approaches are dependent on disease classification. There are six molecular subtypes of the disease, luminal A, luminal B (HER2+ of HER2−), HER2‐enriched, normal‐like and basal‐like or triple‐negative breast cancer (TNBC) (Table 1), which are classified according to the expression of several proteins.^3^, ^4^, ^5^, ^6^

Crucially, expression of these proteins determines which therapies clinicians employ to treat the disease such as neoadjuvant hormone therapies, chemotherapies and/or radiotherapies.^7^, ^8^ Successful treatment outcomes rely on efficient activation of anticancer responses, therefore delineating factors that beneficially or negatively impact associated immune responses are key. One such emerging modulator of BrCa aetiology is the gut microbiota, which may represent a viable therapeutic target for altering the course of the disease.

**TABLE 1:** BrCa subtypes defined by receptor status and proliferative potential as based on Ki67 expression

BrCa Subtype	Receptor status			Ki67 expression	
	ER	PR	HER2		Clinical prognosis
Luminal A	+	+	−	Low	Good
Normal‐like	+/−	+/−	−	Low	Intermediate
Luminal B (HER2−)	+	+/−	−	Any	Intermediate
Luminal B (HER2+)	+	+/−	+	High	Intermediate
HER2‐enriched	−	−	+	Any	Poor
Basal‐like/TNBC	−	−	−	Separate basal markers used, for example, claudin	Poor

## 2 BrCa RISK FACTORS: IS THE MICROBIOTA A MISSING LINK?

Alongside known genetic factors, all cancers are considered to have an “environmental” element associated with increased risk and disease progression. These associations are often uncovered in large human epidemiological studies that correlate lifestyle factors (eg, smoking, drug exposure and diet) with cancer onset and clinical outcomes.^9^, ^10^, ^11^ As many of these factors are also known to alter the gut microbiota, population‐based association studies at least intimate that the gut microbiota dictates cancer outcomes.^9^, ^12^, ^13^ A stable and diverse microbial ecosystem (in adults) is considered optimal for health, although the exact taxa that confer beneficial effects is an ever changing conundrum, and is dependent on age, diet, medications, host genetics and numerous other external factors. In many cases, a reduction in alpha and beta diversity is associated with increased disease risk for many conditions, but correlations with specific microbial taxa may change between patient cohorts. In terms of “beneficial” bacterial members, those best studied include some of the following: *Bifidobacterium*, *Lactobacillus*, *Akkermanisa*, *Ruminococcus*, *Bacteroides* and *Faecalibacterium*; however, species and strain‐level differences are key considerations. More recent studies indicate that overall function of the total microbial community may allow more “healthy” signatures to be found, as recently described for colorectal cancer (CRC) and choline degradation.^14^ For reviews on this subject, see References 15 and 16. The gut‐tumour connection includes sites known to have direct cross talk between the host and the gut microbiota (eg, CRC), but also in sites distal to the gut (eg, the skin, liver and breast). It is likely that, what applies in one cancer setting is by no means universal, and BrCa, by its extreme heterogeneity and relative low incidence of genetic predisposition, is particularly unique. Consequently, there are many large studies focused on understanding how different environmental factors influence BrCa, and each of these factors influence (and are influenced by) the microbiota. These include the following:

1. *Diet*: In the 1990s, several groups investigated the association between diet and BrCa risk. For example, a low‐fat diet elicited a lower risk of relapse after tumour resection.^15^ Recent meta‐analyses of cohort studies continue to correlate dietary patterns with BrCa risk.^16^ This topic is covered in more detail in Section 3 of this review.
2. *Obesity*: Complementary to a low‐fat diet, obesity is associated with increased risk of developing postmenopausal BrCa with a worse clinical outcome. Meta‐analysis of nine studies showed increased BrCa risk with increased body mass index (BMI).^17^ Associations between obesity and postmenopausal BrCa may be due to adipose tissue catalysing the formation of oestrogen after menopause, thereby increasing circulating oestrogen levels^17^, ^18^; see Point (4).
3. *Alcohol consumption*: Excessive alcohol intake is also recognised as a risk factor for BrCa.^19^ Although the specific molecular mechanisms driving this correlation remain unknown, ethanol may (a) induce molecular damage in mammary cells; (b) inhibit oestrogen‐metabolising enzymes in the liver; (c) increase aromatase activity in the liver, which has been reported to facilitate the conversion of testosterone to oestrogen^20^; see Point (4).
4. *Changes in circulating hormonal levels*: Alongside uterine, ovarian and prostate cancers, some forms of BrCa are oestrogen driven. Both a late menarche and an early menopause decrease the risk of developing BrCa.^11^ For a recent review on this subject, see Reference 21.
5. *Antibiotic exposure*: Use of antibiotics is becoming increasingly controversial, with unexpected adverse effects being reported in several disease contexts.^22^, ^23^, ^24^ In 2004, Velicer et al concluded that cumulative days of antibiotic exposure were associated with increased risk of BrCa.^25^ A follow‐up study also showed that antibiotic use may be associated with less favourable tumour features.^26^

One commonality between each of these risk factors is that they significantly alter the profile of the gut microbiota (see Table 2), suggesting a strong link between microbiota make‐up and BrCa development. This suggests that a perturbed microbiota and therefore altered microbial‐associated functions may impact BrCa risk. Generally, in terms of microbiome changes and duration of these alterations in response to different factors, certain “resilient” individuals may have a rapid but only transient change in microbiome profiles, while others may have large‐scale changes that persist over many years (often compounded during the early life window), after introduction and removal of, for example, antibiotics and dietary interventions.^37^ However, this is dependent on the baseline microbiota of individuals, and in many cases we do not understand the impact of these factors on specific microbial communities, species and strains, which may play a key and oversized beneficial role. Further studies are needed in this area, including within BrCa patient cohorts.

**TABLE 2:** BrCa risk factor and microbiota associations

Factor	Influence on the gut microbiota
Diet (covered in more detail in Section 3)	Members of the microbiota can digest otherwise indigestible components of our diet (eg, dietary fibre) Dietary fibre constituents can (a) boost nutritional intake, (b) act as a substrate for other microbiota members to colonise and (c) act as a metabolite^27^ Short‐chain fatty acids (SCFA), a constituent of metabolised dietary fibre, can module host immune responses Bioactive compounds, a constituent of metabolised polyphenols, encourage growth of beneficial bacteria for example, *Bifidobacterium* and *Lactobacillus* and production of SCFA^28^, ^29^
Obesity	Gut microbiota profiles differ among obese and lean patients and between those with metabolic syndrome^30^ In mice, studies showed that an obese microbiota profile had a greater nutritional intake capacity^31^ Members of an obese microbiota profile encoded enzymes that could more efficiently degrade polysaccharides
Alcohol	Perturbations of the gut microbiota profile was observed in alcoholics vs nonalcoholics^32^ This resulted in lower abundance of Bacteroidetes and higher abundance of Proteobacteria Alcoholics also had higher levels of serum endotoxin Alcoholics tended to have greater gut permeability, which could lead to a local inflammatory state and disease, for example, alcohol‐related liver disease^33^ It can be hypothesised that changes in microbiota members due to alcoholism alter the metabolites available by the host to use for other physiological processes including gut barrier function
Hormones	In 1998, a group observed that germ‐free mice, which do not have a gut microbiota, regained normal oestrous levels upon accidental bacterial contamination This suggested a link between gut bacteria and reproductive capacity^34^ Microbiota members possess ß‐glucoronidase, which can deconjugate already metabolised oestrogen Thereby increasing levels of systemic oestrogen, increasing the risk of ER+ breast cancer^35^ A population‐based study demonstrated an association between oestrogen metabolism and phylogenetic diversity of the gut microbiota, suggesting a link between the gut bacteria and circulating reproductive hormones^34^
Antibiotics	Antibiotics severely impact the gut microbiota, most notably they reduce microbial diversity After depletion due to antibiotics it became easier for pathogenic bacteria, for example, *Salmonella* to colonise due to lack of competitive exclusion^36^ The change in microbiota members consequently influenced the availability of metabolites used by the host, which could influence, for example, host immune responses^36^

## 3 GROWING EVIDENCE LINKING THE GUT MICROBIOTA AND BrCa

Changes in the abundance (ie, levels) of particular microbes have been associated with several cancers. A well‐known example is *Helicobacter pylori*, which initiates gastric inflammation and the formation of precancerous lesions.^38^ Likewise, increases in *Fusobacterium* spp. are associated with increased risk of developing CRC.^39^

Functional pathways linking specific microbiota members and BrCa have yet to be shown, but gut microbiota profiling of BrCa patients has indicated that there may be microbial signatures associated with disease stage and outcomes. Microbiota profiling methods and analysis tools are varied and there are important points to be considered when designing or interpreting these types of studies. Microbiota profiling of the Twins UK cohort, which comprises a large number of older women, indicated that BrCa incidence correlated with “disrupted” or a nonhealthy microbiota signature,^40^ suggesting that the gut microbiota may represent a useful biomarker and/or treatment focus for BrCa patients. Studies looking at postmenopausal BrCa patients have shown that these women had an enrichment of 38 species compared to postmenopausal controls, these included species positively (albeit) weakly associated with oestrogen metabolism (*Shewanella putrefaciens* and *Erwinia amylovora*), short‐chain fatty acid butyrate producing species (*Roseburia inulinivorans*) and a species that was negatively but weakly associated with tumour‐infiltrating immune cells (*Actinomyces* sp. HPA0247).^41^ In a different study by Goedert et al, an altered microbiota signature in the same subtype of BrCa patients, that is, postmenopausal BrCa was observed; lower alpha diversity and increased relative abundance in classes Clostridiaceae and Ruminococacceae and in *Faecalibacterium*, and a relative decrease in abundance of *Dorea* and *Lachnospiraceae*.^42^ In short, these studies suggest that postmenopausal BrCa patients do have an altered microbiota signature, which was reported in the Twin UK cohort study.^40^ Guan et al assessed microbiota profiles of HER2 metastatic BrCa patients undergoing metronomic Capecitabine. They observed different microbiota profiles and reduced diversity in Capecitabine vs conventional patients. Further microbiota probing suggested that *Blautia obeum* was significantly associated with progression‐free survival in Capecitabine, while *Slackia* was negatively associated with progression‐free survival.^43^ Wu et al recruited 37 BrCa patients and assessed the microbiota profile based on their tumour characteristics. The group observed that patients who were positive for HER2 had a significantly lower alpha diversity. In addition, they found that patients with a higher tumour grade were associated with a higher abundance of *Clostridium* and *Veillonella*, and a lower abundance of Erysipelotrichaceae.^45^ Both *Veillonella* and Erysipelotrichaeceae have previously been reported to correlate with inflammatory conditions.^44^ In the same study, the group assessed microbiota changes to recognised BrCa risk factors, for example, BMI and menarche. They observed that an early age of menarche was associated with a lower microbiota diversity—which may suggest a link with circulating oestrogen.^44^ Furthermore, larger studies (considering other microbiome confounders) are required to explore patterns/relationships, which may be at the functional level rather than through shared taxa—as recently indicated in CRC (and choline degradation).^14^

Although longitudinal human studies facilitate important insights into the complex relationship between the gut microbiota and BrCa, there are numerous ethical and logistical issues that preclude their use for understanding detailed mechanisms. Thus, in vivo models are crucial to better define the underlying mechanisms driving specific observations under robust controlled conditions.

## 4 ANIMAL STUDIES OF THE LINKS BETWEEN THE GUT MICROBIOTA AND BrCa

To date, there is still a relatively limited body of research exploring the role of the microbiota and different in vivo BrCa models, although interest is growing, and lessons learned from other cancers may be applicable in this underresearched area. Crucially, studies to date have indicated that microbiota modulation of the immune system may represent a key cross‐roads determining disease and treatment outcomes. A distinct advantage of using preclinical models is the ability to explore the impact of microbiota on different BrCa subtypes, which is key given the heterogeneous nature of this cancer type (see Table 1).

### 4.1 Evidence of microbiota involvement in non‐BrCa disease

Understanding mechanistic links between the gut microbiota and BrCa is in its infancy. Thus, to better gauge the potential for the microbiota to influence BrCa occurrence and progression, it is important to consider the larger body of literature confirming such links in non‐BrCa disease. Disruption of gut homeostasis and effects on local inflammatory diseases have been well researched in animal models. Thus, it is unsurprising that CRCs were some of the first to be linked to changes in microbial communities, for example, increased *Fusobacterium nucleatum* abundance has been heavily associated with colorectal carcinogenesis (as mentioned earlier). However, without the use of animal models, it is difficult to conclude whether such changes are causative of disease or simply a product of it. Recently, animal studies have been able to explore these relationships in more detail, for example, Yu et al identified that subcutaneous xenograft tumours, derived from SW480 colon adenocarcinoma cells, intratumorally injected with *F. nucleatum* were resistant to oxaliplatin chemotherapy through an autophagy‐dependent pathway,^46^ suggesting a protumorigenic influence of the bacteria.

Microbes have also been observed to play antitumorigenic roles, which in many cases appears to be via education of the host immune system. One of the seminal studies in the microbiome cancer field identified an association between *Bifidobacterium* abundance and reduced melanoma tumorigenesis in a subcutaneous allograft B16.F10.SIY model in C57/BL6 mice.^47^ The same study observed that oral administration of a cocktail of *Bifidobacterium* species combined with an immune checkpoint inhibitor immunotherapy targeting the PD1—PD‐L1 signalling pathway using an anti‐PD‐1 monoclonal antibody promoted activation of CD8+ cytotoxic T cells and significantly reduced tumour outgrowth.^47^ Tanoue et al presented similar findings when administering mice with a consortium of 11 bacterial strains isolated from healthy human faeces, including those of the genus *Bifidobacterium* and *Lactobacillus*, describing improved CD8 T‐cell activation and improved efficacy of immune checkpoint inhibitor therapies.^48^ Furthermore, Gopalakrishnan et al went on to confirm a similar outcome in human patients, whereby those with an increased microbial diversity responded more favourably to anti‐PD‐1 immunotherapies than patients with lower diversity.^49^

### 4.2 BrCa literature

With the awareness that non‐BrCa disease is influenced by changes in the microbiota, we can now ask if similar affects are observed in BrCa models. Unlike in melanoma, the lower expression of immune‐modulating proteins, such as PD‐1 and CTLA‐4, in BrCa means it is less amenable to checkpoint inhibition therapies.^50^, ^51^ However, the immune system still plays a key role in both pro‐ and anticancer responses at different stages of BrCa progression. In one of the earlier microbiota—BrCa studies, Rao et al observed that *Helicobacter hepaticus* infection in C57/BL6 Apc^Min/+^ mice (spontaneous CRC model) resulted in the formation of tumours in the breast as well as in the colon.^52^ In a similar model deficient in the *Rag2* gene, thus lacking mature lymphocytes, tumours were more frequent with increased infiltration of F4/80^+^ myeloid cells, suggesting a protective role of lymphocytes in reducing innate immune inflammation associated with tumourigenesis. Indeed, dosage of *Rag2*‐deficient animals with CD4^+^CD45^low^CD25^+^ T‐regulatory cells from wild‐type animals significantly reduced breast tumourigenesis, including in *H hepaticus* infected animals.^52^ This supports the consensus that microbes have an integral role in priming the immune system, particularly the adaptive components, to act against cancers distal to the GI tract.

More recent studies have focused on the impact of loss of known mutualistic bacterial genera and species, and the impact of microbial metabolites on BrCa progression (also discussed later). One of the most influential factors contributing to disruption of the gut microbiota is antibiotic use, which has been linked to increased risk of several cancers including BrCa.^53^, ^54^ In a comprehensive study, Buchta Rosean et al administered C57/BL6 mice with a robust antibiotic cocktail, comprising vancomycin, neomycin, metronidazole, gentamycin and ampicillin, for 2 weeks to ablate the gut microbiota.^24^ Following a recolonisation period of 4 days, animals were orthotopically induced with either a poorly metastatic, hormone receptor positive model or a more aggressive PyMT‐derived model. A pre‐perturbed microbiota significantly increased metastasis to the lungs in both models, without influencing primary tumour growth kinetics.^24^ Subsequent analysis of immune cell infiltration and cytokine analysis of mammary tissue prior to tumour induction identified increased abundance of myeloid cells and elevated myeloid recruitment components including CXCL10 and CCL2, suggesting that metastatic potential was promoted through antibiotic‐induced inflammatory pathways independent of tumour status. McKee et al used a similar antibiotic cocktail (swapping gentamycin for amphotericin), but continued treatment throughout the experimental period.^55^ In contrast to Buchta Rosean et al, they did observe a significant increase in primary tumour kinetics when using orthotopic syngeneic models for both luminal (PyMT‐BO1) and basal‐like (EO771) BrCa. Downstream scRNA‐seq revealed an increased stromal signature (PyMT‐BO1 only) and histological analysis revealed increased abundance of mast cells tumour stroma in PyMT‐BO1 and EO771 tumours from antibiotic‐treated animals. Notably, inhibition of mast‐cell activation confirmed this immune population to be driving enhanced primary tumour growth after antibiotic‐induced microbiota disturbances. Importantly, the same study also demonstrated that using a clinically relevant cephalosporin antibiotic promoted the same increase in primary tumour growth and was associated with similar increases in tumour mast cells.^55^

Based on the similarity of the orthotopic models used in these two studies, it is likely that the differences in primary tumour growth between them were due to the differences in treatment regimen. Buchta‐Rosean et al allowed for a bacterial recolonisation period of 4 days, which likely aided in slowing primary tumour growth, possibly through an immunological re‐priming.^24^ However, McKee et al undertook an uninterrupted antibiotic treatment, which may have prevented mutualistic bacteria from recolonising, leaving the primary tumour to grow “unchecked”.^55^ Nonetheless, both studies suggest a healthy microbiota positively regulates antitumour immune pathways. Thus, it is surprising that to date there does not appear to be any mechanistic in vivo studies into whether the administration of beneficial or “probiotic” genera such as *Bifidobacterium* or *Lactobacillus* may influence BrCa progression or support therapeutic intervention against it.

The metabolome of the microbiota is also known to play a key role in host health, influencing an array of biological pathways including cellular proliferation, metabolism and immunity. Although studies focusing on microbial metabolites in BrCa models are very limited, a previous study determined that cadaverine (produced during microbial breakdown of animal tissue) supplementation reduced both primary and metastatic burden in an orthotopic 4T1 triple‐negative‐like BrCa model in BALB/c mice.^56^ As highlighted previously, short‐chain fatty acid (SCFAs, particularly butyrate) also have links to cancer and are known to promote an invasive/aggressive phenotype in BrCa in vitro.^57^ Crucially, these microbial metabolites are derived after fermentation/metabolism of dietary components and therefore diet may act as a key overriding factor that impacts the microbiota, their metabolites and subsequent host interactions, leading to differential BrCa outcomes.

## 5 DIET, THE GUT MICROBIOTA AND BrCa

As already mentioned earlier (and in Table 2), extensive epidemiological studies have laid the groundwork for understanding that diet has a major role on cancer risk and progression.^58^, ^59^, ^60^ One of the key roles played by the microbiota is breakdown of complex dietary substrates into their constituent bioactive compounds; therefore, there is growing interest in understanding functional outcomes and the underlying mechanisms governing diet‐microbe interactions with respect to cancer.

### 5.1 Diet and BrCa

Although the correlations between BrCa risk and dietary intake have been intensively studied, the underlying associations or effector mechanisms remain poorly understood. Historically, increased risk of BrCa has been tied to high intake of red meat and animal fat,^61^, ^62^ with decreased risk being concurrently linked to fruit and vegetables consumption.^63^ The overall field remains conflicted, as epidemiological links between individual foods and BrCa appear difficult to rationalise within the confines of even large‐scale observational studies. This point is emphasised by the 2017 third expert report on “diet, nutrition and physical activity in BrCa” by the World Cancer Research Fund/American Institute for Cancer Research, which stated that although body fatness (as adjudged by BMI and waist‐to‐hip ratio) was a probable risk factor for BrCa in premenopausal and postmenopausal women, there is only limited or suggestive evidence for contribution of any single food group.^64^ It is possible that these findings are a result of the requirement for a change in total dietary pattern to have significant effect on the gut microbiota and disease risk, or because of the difficulty in conducting longitudinal human studies which focus on a single food group. More recently, research has moved toward assessment of dietary patterns (rather than specific foodstuffs), which indicate that “Western” diets (ie, those that are high in processed meat, sugar and fat) increase BrCa risk, while a more “healthy” diet (ie, high fresh fruit, vegetables and fish) decreases BrCa risk.^16^ Importantly, and specific to BrCa, menopausal status and BrCa subtype (ie, receptor status) are important confounders when assessing dietary links. Western diet effects, for example, are only significant in postmenopausal patients with hormone receptor‐positive tumours, while “healthy” diet effects are only significant in premenopausal women, but across receptor‐positive and receptor‐negative tumours.^16^ Alcohol intake is also a significant risk factor, with high consumption linked with disease recurrence and reduced survival.^65^ Another dietary pattern linked to BrCa is the Mediterranean Diet, with recent studies showing an inverse relationship, particularly in the context of triple‐negative disease.^66^, ^67^

### 5.2 Diet and the gut microbiota

There is a strong evolutionary relationship between the gut microbiota and diet. Certain members genomically encode enzymes such as glycoside hydrolases, which allow poly‐ and/or oligosaccharide carbohydrates to be metabolised.^68^ Some “generalist” microbes, such as members of the Bacteroides phyla (eg, *Bacteroides thetaiotaomicron*), degrade a wide array of carbohydrates, while other “specialist” gut microbes (eg, *Rosaburia intestinalis*) degrade specific oligosaccharides.^69^ Ingestion of dietary fibre elicits a dynamic response from communities of these metabolising microbes through extensive primary and secondary degradation. Here, primary degraders (eg, *B. thetaiotaomicron*) convert polysaccharides into oligosaccharides and secondary metabolites (eg, SCFAs), which can then be utilised by secondary degraders (eg, *Eubacterium rectale*) to further enhance nutrient bioavailability (eg, breakdown to monosaccharides) and support community colonisation.^70^ This so‐called “cross‐feeding” is a determinant of gut population dynamics, as increased metabolism often affords selective advantages, which increase microbe abundance and subsequent digestion efficiency.^69^

Previous work in CRC has indicated that microbial‐derived SCFAs, particularly butyrate, have anticancer effects (demonstrated in cancer cell cultures^71^, ^72^ and animal models^73^); however, clinical supplementation studies have proved difficult due to issues with bioavailability and toxicity.^74^ Therefore, a more nuanced microbiota and defined diet approach may represent a more realistic avenue to improve cancer outcomes. This highlights the interlinking relationship between microbiota composition and metabolite production, with both factors requiring consideration if we are to realistically improve cancer outcomes.

More recently, it is now appreciated that specific components from fruit and vegetables (eg, polyphenols) may also be processed and influence the gut microbiota by acting as prebiotics (defined as dietary substrates, which can be utilised by host microorganisms to confer a health benefit).^75^ Dietary polyphenols have well‐documented effects on the host, which has been reviewed elsewhere.^28^ Studies have shown that increased polyphenol intake is associated with higher levels of beneficial bacteria (such as *Bifidobacterium* and *Lactobacillus*) and SCFAs in humans, while also decreasing levels of bacteria that have been associated with disease, so‐called pathobionts.^29^ Polyphenols are known to undergo extensive metabolism via the gut microbiota during conversion to bioavailable metabolites. The magnitude and complexity of these interactions have made detailed studies difficult, but some examples of known biotransformations include *Flavonifractor plautii* conversion of catechin and epicatechin into valerolactones and valeric acids, and soy isoflavones into equol and/or *O*‐desmethylangolensin by *Slackia isoflavoniconvertens* and *Slackia equolifaciens*. Biochemical groups of microbiota‐modulating polyphenols include flavanols (eg, catechin),^76^ resveratrol^77^ and anthocyanins,^78^ which are highly concentrated in foodstuffs such as green tea, berries and red‐wine. While human mechanistic studies involving polyphenols and disease are lacking, the aforementioned polyphenols are able to rescue high‐fat diet (HFD)‐induced microbiota perturbations and elevate murine type II diabetes symptoms through increases in gut *Akkermansia* species.^79^, ^80^, ^81^ Given the emerging importance of the gut microbiota in tumour progression and cancer therapy, dietary manipulation of the system (via dietary fibre and polyphenols) is an important theme to be explored in humans and via mechanistic in vivo studies.

### 5.3 Mechanistic links between diet and BrCa

The majority of in vivo mechanistic knowledge is confined to tumours “local” to the digestive tract (such as gastric, colon and CRC, as discussed earlier). This is perhaps expected, given gastrointestinal tissues come into direct contact with bioactive compounds resulting from digestion. Studies probing the mechanistic impact of diet on BrCa are limited, and most studies to date have explored how HFDs are linked to primary and metastatic tumours. One particular study indicated that an HFD promoted formation of pre‐metastatic niches and lung metastases in mice through activation of myeloid‐derived suppressor cells (MDSC),^82^ but this HFD‐induced metastasis could be significantly ameliorated through treatment with a saponin called glycyrrhizic acid, a plant‐derived phytochemical. Glycyrrhizic acid significantly altered microbiota composition in this context, and concurrently reduced colonic lipopolysaccharide (LPS), NF‐kB and macrophage activity—which correlated with decreases in MDSC infiltration to pre‐metastatic niches. Interestingly, the effect of glycyrrhizic acid was ablated through microbiota depletion with antibiotics, causing re‐introduction of HFD levels of colonic LPS and implicating the gut microbiota as the key effector in this model system. Mechanistic links between diet and BrCa metastasis have also been made elsewhere, as it has been shown the calorie restriction during radiotherapy (for TNBC) causes decreased metastatic burden in mice compared to animals on an ad libitum diet.^83^ This decrease is caused by downregulation of the oncogenic insulin‐like growth factor‐1/Akt pathway—a known regulator of tumour metastasis.^84^

As previously mentioned, dietary polyphenol intake has been linked with improved BrCa outcomes. To date, many of the studies examining the anticancer properties of polyphenols have been performed in vitro, and have often also used unmetabolised purified compounds, therefore reflecting a direct anti‐tumour therapeutic approach.^85^, ^86^, ^87^ Contrastingly, studies exploring dietary polyphenol intake and their downstream effects on BrCa, within the context of “normal digestion”, are thin on the ground. Of the limited mouse studies completed so far, dietary delivery of quercetin was shown to be effective in reducing tumour number and volume in the C3/SV40 Tag model of BrCa.^88^ Elsewhere, oral delivery of grape polyphenols (resveratrol, catechins and quercetin) decreased primary tumour growth and metastases in a mouse xenograft model via downregulation of NFkB,^89^ and oral piceatannol administration decreased 4T1 breast tumour metastasis through inhibited macrophage infiltration and angiogenesis.^90^

Although it is clear that diet, and specific components like fibre and polyphenols, may play an important role in BrCa, comprehensive studies directly linking the gut microbiota, dietary components and BrCa progression are required to fully understand how we can manipulate the system.

## 6 FUTURE PERSPECTIVES

The field of microbiome and cancer is rapidly expanding, yet studies focused on BrCa are currently limited. There is now a strong body of evidence indicating the gut microbiota and associated factors such as diet and antibiotics may play a key role in BrCa risk and outcomes, therefore detailed mechanistic studies in preclinical models that help underpin next‐stage translational projects are needed. However, there are some important considerations specifically in relation to BrCa that need to be evaluated.

One such consideration relates to the differential risks between BrCa subtypes (see Table 1), including treatment‐resistant (eg, TNBC) tumours. To date this fundamental BrCa factor has been somewhat ignored, therefore future microbiota profiling studies of BrCa should consider clear stratification of patients by histological and molecular subtypes, which may allow comparison between groups and associated pathological outcomes. Age will also need to be carefully considered in these groups as the microbiota diversity does decrease in elderly populations.^91^ Previous studies have indicated that healthy individuals living at home have a more “robust” microbiota compared to those living in long‐term residential care (which may be linked to diet). There are also alterations in microbiome composition associated with frailty indices and inflammatory status.^92^ This is obviously important for BrCa as, the older a woman is, the more likely she is to get BrCa; rates are highest in women over 70.^10^ Furthermore, to date, nearly all BrCa microbiome studies have focused on high‐income country patient populations. As BrCa rates are increasing in low‐to‐middle income countries, which also have differing patient population characteristics (age and BrCa subtype),^93^ further studies should establish signatures in diverse BrCa patient cohorts as this is crucial for next‐stage clinical trials and optimal patient outcomes. An important consideration for microbiome and BrCa studies also relates to standard clinical care pathways. Typically, BrCa patients will undergo one or more of the following: surgery, chemotherapy and/or radiotherapy. Several studies are currently ongoing (either still recruiting or still to publish results) to determine the impact of chemotherapy and the gut microbiota on BrCa patients, for example, NCT03702868, NCT04138979 or NCT03222856; however, findings are yet to be published. Conversely, there appears to be no studies that are solely focused on the relationship between radiotherapy in BrCa patients and its impact on the gut microbiota and clinical outcomes, although there appears to be studies with a radiotherapy aspect (in combination with other therapies, that is, immune‐checkpoint) but not as a primary outcome (NCT04435964). Interestingly, in vivo studies from the 1960s have suggested that radiotherapy on germ‐free mice was less effective^94^; however, further studies—mechanistic and clinical—are required to understand the influence of these differing factors on the gut microbiota and BrCa outcomes.

Although the limited studies to date have indicated an altered microbiota signature in BrCa patients, further research into the strain‐level variation and functional capacity of the microbiota, including a focus on nonbacterial members, that is, viruses, fungi and archaea, and also impact of external factors is required. This may allow more specific biomarker profiling in relation to BrCa risk in relation to the many microbiota‐modulating factors earlier in life may predispose to onset of cancer, including BrCa. Thus, longer‐term longitudinal in‐depth profiling studies are key to understanding the impact of lifestyle factors and routine medications (eg, antibiotics) on the microbiota prior to detection of primary breast tumours. Enhanced microbiota profiling studies may also allow patient stratification for treatment trials (identifying potentially nonresponsive patients) in tandem with current or new therapies (as described earlier). Moreover, more robust microbiome‐profiling approaches and tools are expected to facilitate development and interventions/treatment with microbiota therapies such as probiotics and/or live biotherapeutic products. Probiotics are products that contain live microorganisms, which when administered in adequate amounts can confer health benefits to the host.^95^ Certain types of these probiotic microbes have been shown to stimulate immune responses and also shown in vitro to exert antitumorigenic potential via immunoregulatory pathways.^96^ However, to date, there has only been a very small number of clinical trials to evaluate probiotic supplementation in BrCa patients: NCT03760653 and NCT03358511. Unfortunately, the former was prematurely terminated, while the latter has recently completed with only 7 participants enrolled. It is clear that larger‐scale randomised placebo controlled trials are needed, but ideally these should be based on evaluation of probiotics or live biotherapeutic products in vitro and in vivo to pinpoint potential multifactorial mechanisms underlying beneficial effects, and thus optimal strain(s) combined to take forward into BrCa patients. For a recent review on this topic, see Reference 96.

As diet is a significant confounder for all microbiota studies, and the apparent close links between diet and BrCa, food diaries (using new apps) may provide important insight into the patient's dietary habits. However, current dietary patterns may not reflect previous eating habits, which may have contributed to disease development or susceptibility. Future observational or clinical trials should seek to control, or capture associated meta‐data, which will be crucial for teasing out causative or associative factors with respect to BrCa risk. Moreover, studies focused on diet/metabolites may also need to consider if dietary interventions should be personalised if they are to be successfully utilised for future therapy. Indeed, recent studies have highlighted the personalised response to individuals (and the microbiota) to the same diet,^97^ which highlights the limitations and challenges for next‐stage studies of this kind.

It is clear that more comprehensive preclinical data are required in the field, and that choice of in vivo models is key. Consideration of the models used, their clinical relevance, microbiota modulatory factors (eg, diet and antibiotics) and the approaches used to integrate microbial and host components must be carefully reviewed. Although subcutaneous tumour studies demonstrate to some extent that microbes can influence carcinogenesis, it is beneficial to test such hypotheses in more physiologically relevant orthotopic or spontaneous models, which better underpin the physiological and biomolecular mechanisms involved in such phenotypes. Indeed, spontaneous in vivo BrCa models may allow further insights into microbiome and microbiota‐modulating factors and BrCa risk, while orthotopic models may allow testing of acute immune mechanisms and testing of new promising therapies. The greater translational impact of these models should provide greater clarity for moving preclinical research to human studies. Moreover, studies focusing on dietary or antibiotic interventions in mouse models should include downstream analyses of the gut microbiota, mucosal and systemic immunity, as well as microbial and host metabolism, so to develop a multisystem picture of the effects of microbiota manipulations on BrCa risk and progression. An important caveat to any resulting findings, however, is that mice of the same genetic background housed at different animal facilities will likely have different microbiota profiles, which may influence experimental cancer outcomes.^98^ Overall, more comprehensive preclinical findings will better inform and facilitate design of translational human studies looking at the impact of defined dietary components on the gut microbiota and BrCa outcomes, as well as profiling responders vs nonresponders to treatments in the context of diet.

## 7 CONCLUSIONS

The microbiome is emerging as a central player in cancer risk and anticancer responses, and recent studies also suggest this may be the case for BrCa. However, as many intrinsic and extrinsic factors are known to influence the microbiota, and indeed the cancer itself may also impact community composition, robust experimental design from sequencing the microbiota to capturing meta‐data, and the choice of in vivo models, is required. A combination of approaches is most likely to narrow down important causative or associative factors, which may allow interventions to reduce BrCa risk, and guide the development of novel therapeutic approaches to alleviate symptoms or improve prognosis in BrCa patients.

## CONFLICT OF INTEREST

The authors declare no conflicts of interest.
